# Supplementary material for: Comparison of Primary Virus Isolation in Pulmonary Alveolar Macrophages and Four Different Continuous Cell Lines for Type 1 and Type 2 Porcine Reproductive and Respiratory Syndrome Virus
Source: Vaccines (Basel). 2021 Jun 3;9(6):594. doi: 10.3390/vaccines9060594 (PMC8229515; doi:10.3390/vaccines9060594)
Supplement: Supplementary file 1 [file vaccines-09-00594-s001.zip › vaccines-1220358-supplementary.pdf]

vaccines

MDPI

Table S1. Titration results for PRRSV1 on PAM, $PK15^{Sn-CD163}$, $PK15^{S10-CD163}$, MARC-145^{Sn} and MARC-145.

No.	Serum sample number sample	Sequence availability ence	Virus titers on …. cells (log_{10}/mL)				
			PAM	$PK15^{Sn-CD163}$	$PK15^{S10-CD163}$	$MARC^{Sn}$	MARC
1	18v195-1	full	2.8	1.6	<1[b]	<1	<1
2	18v195-2	na[a]	<1	<1	<1	<1	<1
3	18v195-3	na	<1	<1	<1	<1	<1
4	18v195-4	full	2.3	1.0	1.0	<1	<1
5	18v195-5	na	<1	<1	<1	<1	<1
6	18v195-6	na	<1	<1	<1	<1	<1
7	18v195-7	full	1.8	<1	<1	<1	<1
8	18v195-8	full	3.6	2.1	<1	<1	<1
9	18v195-9	na	<1	<1	<1	<1	<1
10	19v13-1	full	4.5	3.3	3.0	3.6	2.8
11	19v13-2	na	<1	<1	<1	<1	<1
12	19v13-3	full	4.5	3.2	2.3	<1	<1
13	19v13-4	full	2.6	<1	<1	<1	<1
14	19v13-5	na	<1	<1	<1	<1	<1
15	19v13-6	partial	2.9	1.0	1.0	<1	<1
16	19v13-7	full	2.0	<1	<1	<1	<1
17	19v13-8	na	<1	<1	<1	<1	<1
18	19v13-9	na	<1	<1	<1	<1	<1
19	19v13-10	na	<1	<1	<1	<1	<1
20	1901-1055	na	<1	<1	<1	<1	<1
21	19v93-2	full	2.3	<1	2.0	2.0	2.1
22	1901-1058	na	2.0	<1	<1	<1	<1
23	1901-1100	na	<1	<1	<1	<1	<1
24	19v93-5	full	3.1	2.6	2.3	<1	<1
25	19v93-6	partial	2.0	2.0	<1	<1	<1
26	1901-1166	na	<1	1.6	1.6	<1	<1
27	1901-1172	na	<1	<1	<1	<1	<1
28	1902-1043	na	<1	<1	<1	<1	<1
29	1902-1044	na	<1	<1	<1	<1	<1
30	1902-1065	na	<1	<1	<1	<1	<1
31	19v93-12	full	1.1	1.0	1.3	<1	<1
32	19v93-13	full	<1	<1	<1	1.8	1.1
33	19v93-15	full	<1	1.3	<1	1.0	2.5
34	19v93-17	full	3.3	2.3	1.5	<1	1.1
35	1904-1027	na	<1	<1	<1	<1	<1
36	1904-1028	na	<1	<1	<1	<1	<1
37	19v93-21	full	1.3	<1	<1	<1	<1
38	19v93-22	full	1.0	1.0	<1	<1	<1
39	19v93-23	full	2.8	3.3	2.0	<1	2.0
40	19v105-930	na	<1	<1	<1	<1	<1
41	19v105-931	na	<1	<1	<1	<1	<1
42	19v105-933	full	2.0	<1	<1	<1	<1
43	19v105-950	full	4.5	3.3	3.0	<1	<1
44	19v105-951	full	4.5	4.5	3.0	2.0	2.8
45	19v105-952	full	4.5	3.3	2.6	<1	<1
46	19v105-966	full	1.5	<1	<1	<1	<1
47	19v105-967	na	<1	<1	<1	<1	<1

[a]na: not available; [b]<1 : detection limit.

Table S2. Titration results for PRRSV2 on PAM, PK15$^{Sn-CD163}$, PK15$^{S10-CD163}$, MARC-145^{Sn} and MARC-145.

No.	Serum Sample	Sequence	Virus titers on … cells (log_{10}/mL)				
			PAM	PK15$^{Sn-CD163}$	PK15$^{S10-CD163}$	MARC^{Sn}	MARC
1	1949752	partial	2.6	<1 [a]	<1	<1	<1
2	1953024	full	4.5	2.0	<1	2.0	<1
3	1966316	full	2.3	1.3	1.6	<1	<1
4	1982260	full	3.6	<1	<1	<1	<1
5	1982261	partial	2.7	<1	<1	<1	<1
6	1982353	full	4.5	4.5	3.3	1.6	2.0
7	1982354	full	4.5	4.5	3.6	2.6	2.0
8	1985928	full	4.6	4.5	4.5	3.0	3.0
9	1989513	full	3.0	<1	<1	<1	<1
10	2010362	full	<1	<1	<1	<1	<1
11	2018114	partial	<1	<1	<1	<1	<1
12	2018119	full	<1	2.0	<1	<1	<1
13	2019713	full	<1	<1	<1	<1	<1
14	2029069	full	3.3	2.8	3.0	2.0	<1
15	2035290	full	2.6	1.3	1.0	2.6	2.0
16	2048380	full	3.3	<1	1.3	2.3	<1
17	2050887	full	2.0	1	<1	2.3	2.3
18	2053682	full	3.3	3.6	3.6	2.3	3.3
19	2057423	full	2.0	<1	<1	<1	<1
20	2067408	full	<1	1.0	1.0	3.0	4.5
21	2073979	full	3.6	2.6	2.6	2.6	3.0
22	2078279	full	1	<1	<1	1.6	1.0
23	2081331	partial	3.4	2.1	2.0	2.6	3.5
24	2087622	full	1.8	<1	1.0	<1	<1
25	2092399	full	3.7	2.3	2.0	2.0	2.8
26	2101687	full	3.6	2.3	1.3	<1	<1
27	2104407	full	4.5	4.5	3.6	<1	<1
28	2108625	full	3.0	2.3	<1	2.0	2.0
29	2109640	full	3.1	3.3	2.3	3.0	3.0
30	2154774	full	4.6	4.5	3.0	3.0	2.8

[a] <1 : detection limit.

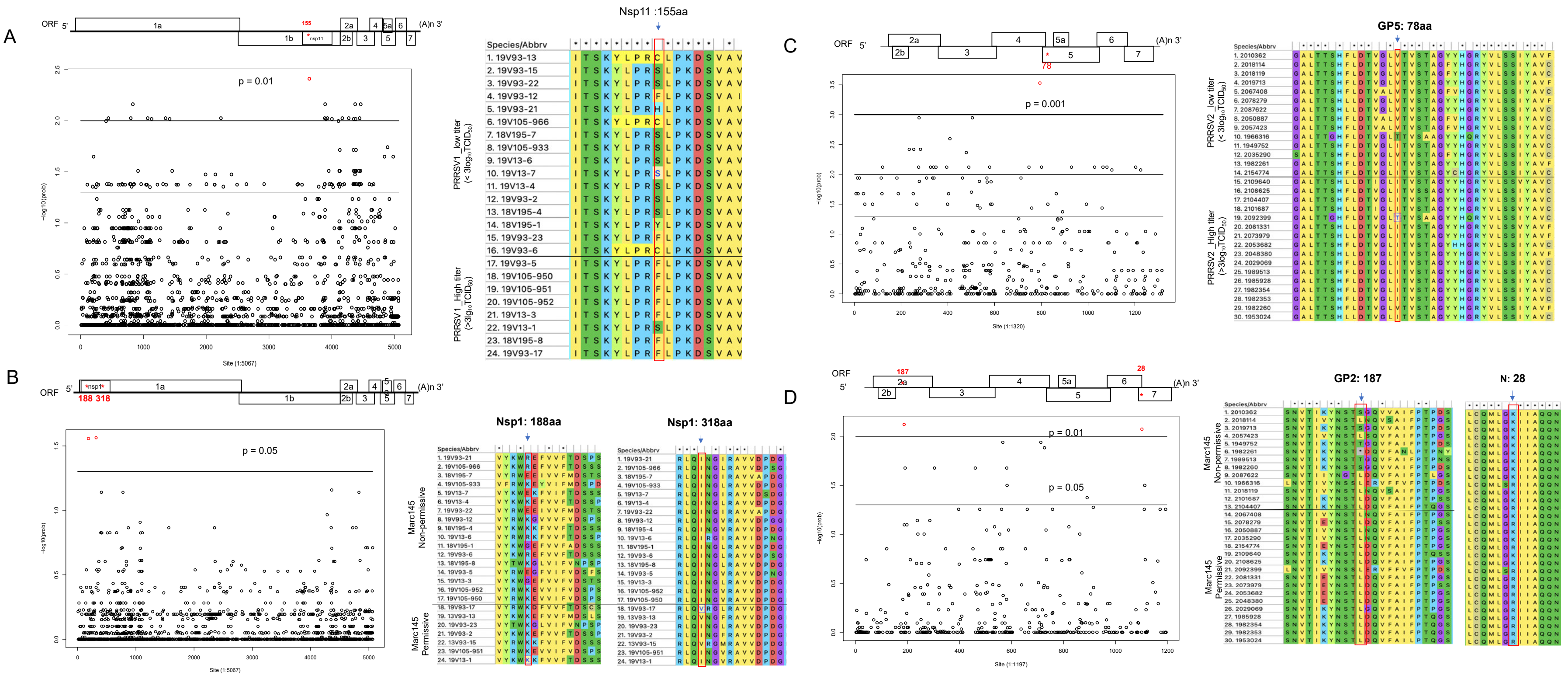

Figure S1. Results of GWAS shows potential residues linked to the cell tropism both for PRRSV1 and PRRSV2. A. PRRSV1 sequence comparison between the isolates with high vs. low titers (on PAM). B. PRRSV1 sequence comparison between MARC-145 permissive isolates and non-permissive isolates. C. PRRSV2 sequence comparison between the isolates with high vs. low titers (on PAM). D. PRRSV2 sequence comparison between MARC-145 permissive and non-permissive isolates. Residues showed a significant difference ($p < 0.05$ and/or $p < 0.01$) between the compared groups were indicated with the position in the corresponding viral protein. The GWAS analysis was performed with the custom R script (https://github.com/itrus/GWAS-fasta) [40]. Sequence alignment for the identified residues were performed and analyzed with MEGA6.
